# Skeletal muscle mitochondrial inertia is associated with carnitine acetyltransferase activity and physical function in humans

**DOI:** 10.1172/jci.insight.163855

**Published:** 2023-01-10

**Authors:** Rodrigo F. Mancilla, Lucas Lindeboom, Lotte Grevendonk, Joris Hoeks, Tim R. Koves, Deborah M. Muoio, Patrick Schrauwen, Vera Schrauwen-Hinderling, Matthijs K.C. Hesselink

**Affiliations:** 1 NUTRIM School of Nutrition and Translational Research in Metabolism and; 2 Department of Nutrition and Movement Sciences, Maastricht University Medical Center, Maastricht, Netherlands.; 3 Duke Molecular Physiology Institute and Sarah W. Stedman Nutrition and Metabolism Center, Duke University Medical Center, Durham, North Carolina, USA.; 4 Department of Radiology, Maastricht University Medical Center, Maastricht, Netherlands.

**Keywords:** Aging, Metabolism, Diabetes, Mitochondria, Skeletal muscle

## Abstract

**BACKGROUND:**

At the onset of exercise, the speed at which phosphocreatine (PCr) decreases toward a new steady state (PCr on-kinetics) reflects the readiness to activate mitochondrial ATP synthesis, which is secondary to Acetyl-CoA availability in skeletal muscle. We hypothesized that PCr on-kinetics are slower in metabolically compromised and older individuals and are associated with low carnitine acetyltransferase (CrAT) protein activity and compromised physical function.

**METHODS:**

We applied ^31^P-magnetic resonance spectroscopy (^31^P-MRS) to assess PCr on-kinetics in 2 cohorts of volunteers. Cohort 1 included patients who had type 2 diabetes, were obese, were lean trained (VO_2_max > 55 mL/kg/min), and were lean untrained (VO_2_max < 45 mL/kg/min). Cohort 2 included young (20–30 years) and older (65–80 years) individuals with normal physical activity and older, trained individuals. Previous results of CrAT protein activity and acetylcarnitine content in muscle tissue were used to explore the underlying mechanisms of PCr on-kinetics, along with various markers of physical function.

**RESULTS:**

PCr on-kinetics were significantly slower in metabolically compromised and older individuals (indicating mitochondrial inertia) as compared with young and older trained volunteers, regardless of in vivo skeletal muscle oxidative capacity (*P* < 0.001). Mitochondrial inertia correlated with reduced CrAT protein activity, low acetylcarnitine content, and functional outcomes (*P* < 0.001).

**CONCLUSION:**

PCr on-kinetics are significantly slower in metabolically compromised and older individuals with normal physical activity compared with young and older trained individuals, regardless of in vivo skeletal muscle oxidative capacity, indicating greater mitochondrial inertia. Thus, PCr on-kinetics are a currently unexplored signature of skeletal muscle mitochondrial metabolism, tightly linked to functional outcomes. Skeletal muscle mitochondrial inertia might emerge as a target of intervention to improve physical function.

**TRIAL REGISTRATION:**

NCT01298375 and NCT03666013 (clinicaltrials.gov).

**FUNDING:**

RM and MH received an EFSD/Lilly grant from the European Foundation for the Study of Diabetes (EFSD). VS was supported by an ERC starting grant (grant 759161) “MRS in Diabetes.”

## Introduction

Regular physical activity is a major contributor to metabolic health. In light of an aging and predominantly sedentary population, the incidence of exercise intolerance has grown over the past decades (1). Of note, exercise intolerance typically manifests as premature muscle exhaustion upon objective measures of contractile performance (2), and it is associated with poor metabolic health (3). To take advantage of the health benefits of exercise, it is crucial to better understand the origin of exercise intolerance.

At the onset of exercise, phosphocreatine (PCr) rapidly buffers the sudden increase in energy demand. PCr decreases until a new steady state is reached during exercise, when contractile activity is maintained by alternative sources of ATP synthesis, a fundamental process to prevent full PCr depletion and premature muscle fatigue (4). At low-moderate–intensity exercise, ATP is mainly produced by mitochondrial oxidative phosphorylation (OXPHOS). Given the importance of PCr as an ATP source at the onset of exercise and the role of mitochondrial ATP synthesis in reaching and maintaining the new steady state of PCr, the time that it takes to reach a steady state during exercise (here referred to as PCr on-kinetics) reflects the readiness to activate mitochondrial ATP synthesis upon a sudden increase in ATP demand at the beginning of exercise (5) (also referred to as mitochondrial inertia; ref. 6). Thus, skeletal muscle mitochondrial inertia may be a determinant of skeletal muscle physical performance and functional decline. In fact, the occurrence of functional decline (and associated exercise intolerance) has been shown to be higher in metabolically compromised and older sedentary individuals as compared with healthy and physically active peers (7). In line with this reasoning, we anticipate PCr on-kinetics to be slower in individuals with type 2 diabetes mellitus (T2DM) than in healthy individuals, and similarly, we expect slower PCr on-kinetics in sedentary versus physically active older individuals.

The activation of skeletal muscle mitochondrial metabolism at the onset of exercise is secondary to intramyocellular Acetyl-CoA availability (6). Acetyl-CoA availability largely depends on glycolysis and fatty acid oxidation, but a short-term Acetyl-CoA buffer is represented by the intracellular acetylcarnitine pool, which can be converted to Acetyl-CoA by the mitochondrial enzyme carnitine acetyltransferase (CrAT) (8). Interestingly, we previously reported that CrAT protein activity and acetylcarnitine content in human skeletal muscle were significantly lower in patients with T2DM and individuals with obesity (OB) as compared with endurance-trained people, supporting the notion that reduced CrAT protein activity — and therefore a low capacity to form acetylcarnitine from Acetyl-CoA and carnitine — might underlie metabolic inflexibility and impaired insulin sensitivity (9). Importantly, the CrAT enzyme also functions in the reverse direction to supply Acetyl-CoA from acetylcarnitine when energy demand suddenly increases. We here hypothesize that CrAT activity and acetylcarnitine content in skeletal muscle affect mitochondrial inertia and are determinants of PCr kinetics at the onset of exercise. This hypothesis was tested in 4 groups of volunteers including: (a) patients with T2DM, (b) normoglycemic individuals with OB, (c) young lean untrained (UT; VO_2_max < 45 mL/kg/min), and (d) young lean endurance trained (T; VO_2_max > 55 mL/kg/min). To examine the putative functional relevance of slow PCr on-kinetics, we included a second cohort of young (20–30 years) and older (65–80 years) volunteers, with a wide range in skeletal muscle physical function as determined by functional markers of physical fitness in daily life (6-minute walk test [6MWT] and a chair-stand test) and exercise efficiency.

## Results

### Participant characteristics.

Participant characteristics from study cohort 1 are shown in Table 1. Patients with T2DM and OB were significantly older and had a higher body weight, BMI, and fat mass (%) than T and UT counterparts (*P* < 0.05 for all comparisons). Patients with T2DM and OB individuals exhibited significantly lower whole-body insulin sensitivity as compared with T and UT volunteers (M value, *P* < 0.001). Maximal aerobic capacity (VO_2_max) was significantly higher in T as compared with UT individuals and those with OB and T2DM (*P* < 0.001 for all comparisons).

### Skeletal muscle mitochondrial inertia at the onset of exercise in individuals with T2DM and OB.

**Figure 1:**
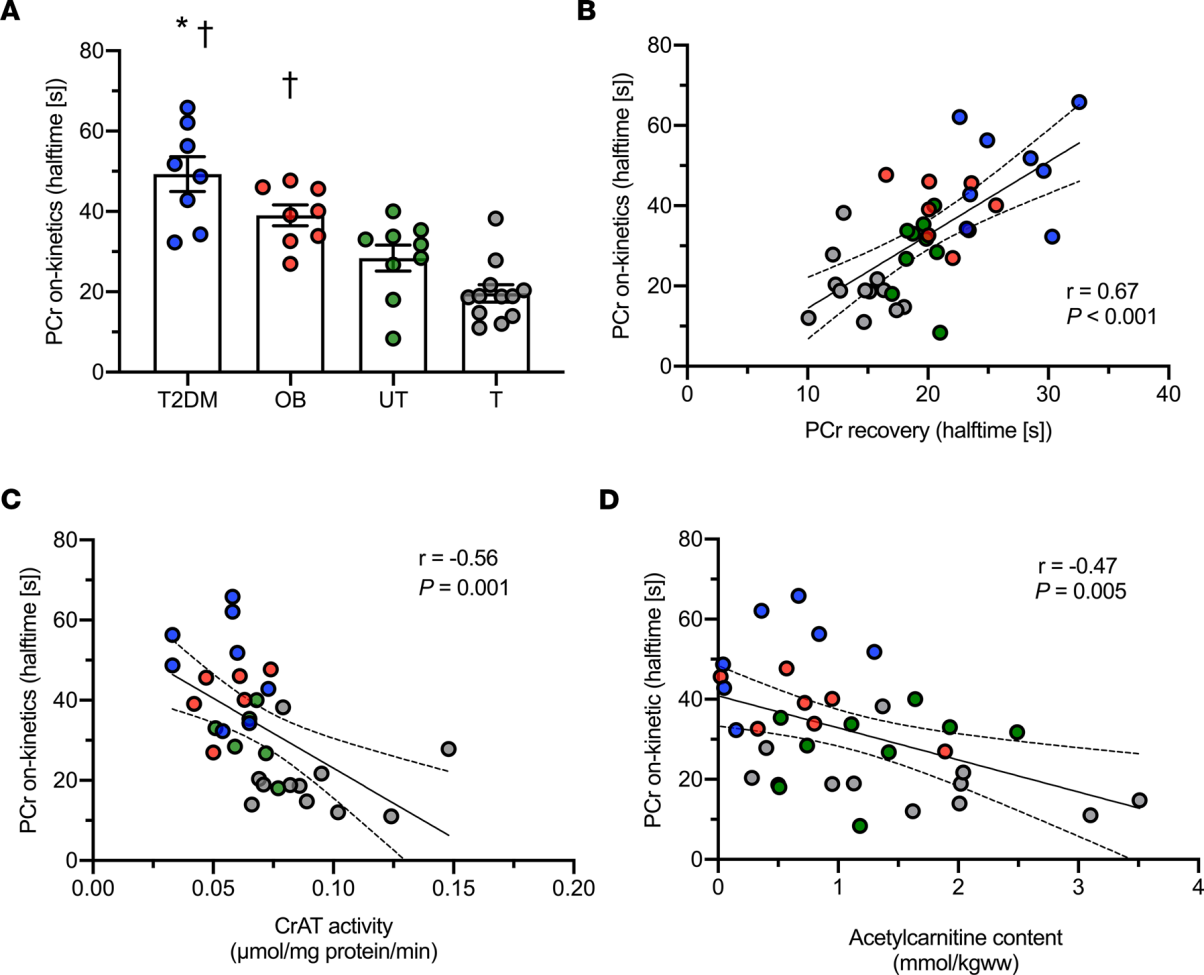
Pronounced skeletal muscle mitochondrial inertia in metabolically compromised individuals. (**A**) Halftime of PCr on-kinetics. (**B**) Linear association between PCr on-kinetics and PCr recovery after exercise. (**C**) Linear association between PCr on-kinetics and CrAT protein activity. (**D**) Linear association between PCr on-kinetics and in vivo skeletal muscle acetylcarnitine content. [s], seconds; T2DM, patients with type 2 diabetes; OB, individuals with obesity; UT, lean untrained; T, trained. Circles in blue, T2DM (*n* = 8); circles in red, OB (*n* = 8); circles in green, UT (*n* = 11); circles in gray, T (*n* = 12). Data are shown as individual points and mean ± SEM. * *P* < 0.05 versus untrained individuals, †*P* < 0.05 versus trained individuals. PCr on-kinetics could not be measured in one T2DM patient due to unreliable steady state and, therefore, are excluded for the analysis. Statistical test were 1-way ANOVA with Bonferroni post hoc correction and Pearson’s correlation.

PCr halftime at the onset of exercise was significantly different across the groups (T2DM, 49.2 ± 4.3 s; OB, 38.9 ± 2.6 s; UT, 28.3 ± 3.2 s; and T, 19.6 ± 2.1 s; *P* < 0.001; Figure 1A). Post hoc analysis revealed that PCr on-kinetics were significantly slower in individuals with T2DM and OB as compared with T volunteers (*P* < 0.001 for both comparisons). Furthermore, PCr on-kinetics were significantly slower in patients with T2DM as compared with UT individuals (*P* < 0.001).

We previously reported (9) that in vivo skeletal muscle mitochondrial capacity, as determined by the rate constant of PCr recovery after exercise, was significantly different across the groups (*P* < 0.001), with a significantly slower PCr recovery in patients with T2DM as compared with those with OB or UT and T individuals (*P* = 0.04, *P* < 0.01, and *P* < 0.01, respectively). Also, PCr recovery after exercise was significantly slower in T2DM patients as compared with those with OB and UT individuals (*P* < 0.005). In order to investigate whether a slow PCr on-kinetics simply reflects lower mitochondrial function, we adjusted the PCr on-kinetics for PCr recovery after exercise. Of note, the significant differences in PCr on-kinetics observed in the current study between patients with T2DM and T and UT individuals remained, even upon correction for PCr recovery after exercise (*P* = 0.006 and *P* = 0.010 upon correction for PCr recovery). The significant difference in PCr on-kinetics between individuals with OB and T individuals also remained, upon correction for PCr recovery after exercise (*P* = 0.010). Furthermore, PCr on-kinetics (mitochondrial inertia) and PCr recovery rate after exercise were strongly associated (*n* = 37, *r* = 0.67, *P* < 0.001; Figure 1B).

### Reduced skeletal muscle CrAT protein activity and low acetylcarnitine content in individuals with T2DM and OB are related to skeletal muscle mitochondrial inertia.

We previously published that skeletal muscle CrAT protein activity was significantly lower in patients with T2DM or OB and UT individuals, as compared with T volunteers (*P* < 0.01), whereas skeletal muscle acetylcarnitine content was significantly lower in patients with T2DM as compared with T individuals (*P* = 0.017) (9). In the current study, we tested if skeletal muscle CrAT protein activity and acetylcarnitine content was associated with PCr on-kinetics. Interestingly, we observed strong negative correlations between both skeletal muscle CrAT protein activity (*n* = 31, *r* = –0.56, *P* = 0.001) and in vivo skeletal muscle acetylcarnitine content (*n* = 35, *r* = –0.47, *P* = 0.005) with PCr on-kinetics halftime (Figure 1, C and D). Interestingly, the association between in vivo skeletal muscle acetylcarnitine content and PCr on-kinetics halftime remained significant after adjusting for the PCr recovery after exercise (*r* = –0.40, *P* = 0.034). In contrast, the association between CrAT protein activity and PCr on-kinetics halftime was not significant after adjusting for PCr recovery after exercise (*r* = –0.17, *P* = 0.37).

### Skeletal muscle mitochondrial inertia is associated with elevated ADP levels in muscle of metabolically compromised individuals.

**Figure 2:**
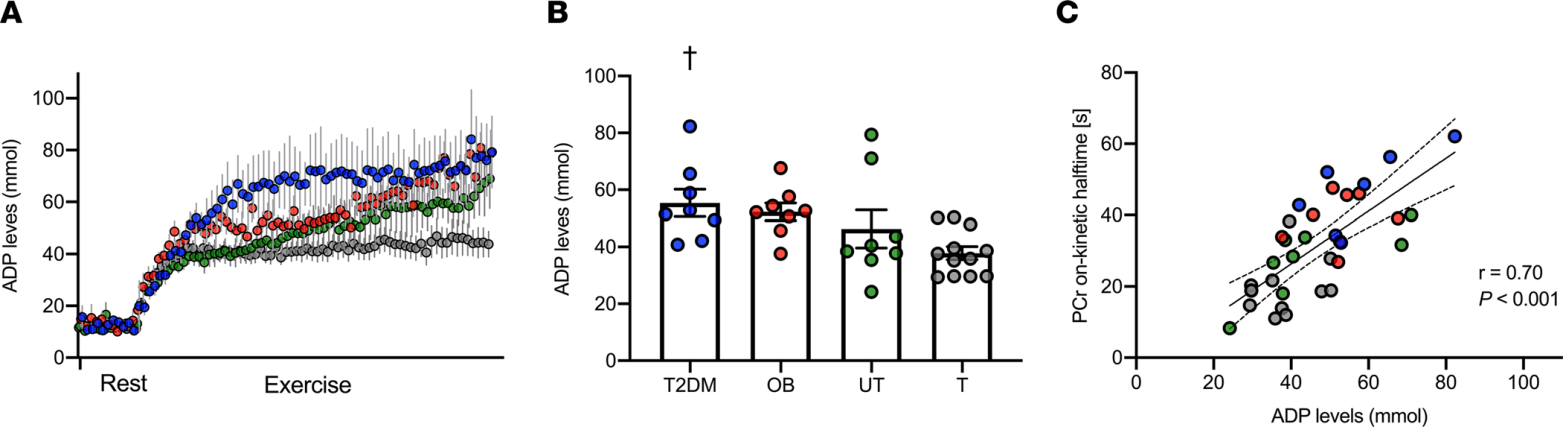
Skeletal muscle mitochondrial inertia coexists with elevated ADP accumulation in muscle tissue during exercise. (**A**) ADP levels in muscle tissue increases upon exercise onset. (**B**) ADP levels when PCr utilization reaches a new steady state at the onset of exercise. (**C**) Linear association between PCr on-kinetics and ADP levels when PCr reached a new steady state during exercise. [s], seconds; T2DM, patients with type 2 diabetes; OB, individuals with obesity; UT, lean untrained; T, trained. Circles in blue, T2DM (*n* = 8); circles in red, OB (*n* = 7); circles in green, UT (*n* = 8); circles in gray, T (*n* = 12). Data are shown as individual points and mean ± SEM. †*P* < 0.05 versus trained volunteers. Statistical test were 1-way ANOVA with Bonferroni post hoc correction and Pearson’s correlation.

Prior to exercise, ADP levels (μM) at rest were similar across the groups. At the onset of exercise, ADP levels increased rapidly in all individuals (Figure 2A). When PCr reached a new steady state during exercise, ADP levels were significantly different across the groups (patients with T2DM, 55.4 ± 4.7 μM; OB individuals, 52.3 ± 3.0 μM; UT individuals, 46.3 ± 6.6 μM; T individuals, 37.8 ± 2.2 μM; *P* = 0.018; Figure 2B), with significantly higher levels in patients with T2DM as compared with T volunteers (*P* = 0.025). When PCr reached a new steady state during exercise, ADP levels were significantly associated with PCr on-kinetics (*n* = 35, *r* = 0.70, *P* < 0.001; Figure 2C). Interestingly, this association remained significant after adjusting for the PCr recovery after exercise (*r* = 0.61, *P* < 0.001). Intracellular pH was not significantly different across the groups, neither at rest (patients with T2DM, 7.1 ± 0.02; OB individuals, 7.1 ± 0.03; UT individuals, 7.1 ± 0.01; T individuals, 7.1 ± 0.02; *P* = 0.90) nor at the end of exercise (patients with T2DM, 7.1 ± 0.02; OB individuals, 7.1 ± 0.04; UT individuals, 7.02 ± 0.03; and T individuals, 7.00 ± 0.02; *P* = 0.08).

Subsequently, we sought to determine whether PCr on-kinetics — hence, skeletal muscle mitochondrial inertia — are related to age, training status, and functional capacities in a second cohort consisting of young (Y) and older (O) individuals with normal physical activity, as well as trained older adults (OT). The characteristics of study cohort 2 are shown in Table 2.

### Skeletal muscle mitochondrial inertia at the onset of exercise in older individuals is rescued by exercise training.

**Figure 3:**
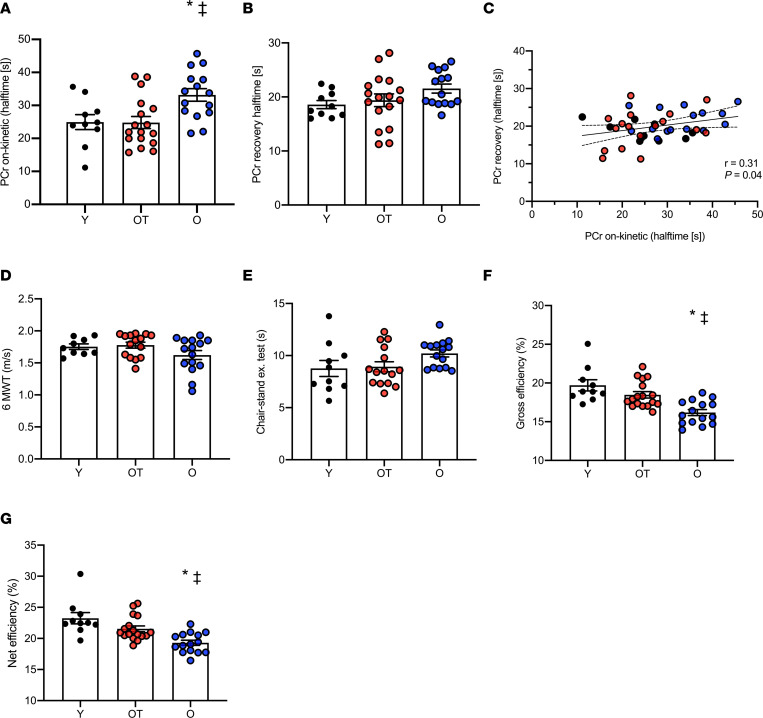
Skeletal muscle mitochondrial inertia and reduced functional outcomes in older sedentary individuals. (**A**) Halftime of PCr on-kinetics. (**B**) Halftime of PCr recovery after exercise. (**C**) Linear association between PCr on-kinetics and PCr recovery after exercise. (**D**) Walking speed upon performing the 6-minute walking test (6MWT). (**E**) Seating and standing upon performing the chair-stand test. (**F**) Gross exercise efficiency. (**G**) Net exercise efficiency upon performing a submaximal cycling test. [s], seconds; Y, young; OT, older trained; O, older with normal physical activity. Circles in black, Y (*n* = 10); circles in red, OT (*n* = 17); circles in blue, O (*n* = 15). Data are shown as individual points and mean ± SD. ^‡^*P* < 0.05 versus Y individuals, *P < 0.05 versus OT individuals. One participant from Y and 2 participants from OT did not perform the 6MWT due to scheduling issues. Two other participants from OT did not perform the chair-stand test due to scheduling issues. Statistical test were 1-way ANOVA with Bonferroni post hoc correction and Pearson’s correlation.

In line with our findings from individuals of cohort 1, PCr on-kinetics were significantly different across groups (Y, 24.9 ± 2.2 s; OT, 24.7 ± 1.8 s; O, 33.1 ± 1.8 s; *P* = 0.002; Figure 3A) with significantly longer values for the halftime in O individuals as compared with OT (*P* = 0.005) and Y individuals (*P* = 0.02). Of note, PCr on-kinetics were not significantly different between OT and Y groups (*P* > 0.05). PCr recovery halftime after exercise was not significantly different across groups (Y, 18.5 ± 0.7 s; OT, 19.3 ± 1.1 s; O, 21.5 ± 0.8 s; *P* = 0.133; Figure 3B). Similar to our findings in cohort 1, PCr on-kinetics (mitochondrial inertia) and PCr recovery halftime after exercise (in vivo mitochondrial function) were significantly associated (*n* = 42, *r* = 0.31, *P* = 0.04; Figure 3C). Interestingly though, the significant difference in PCr on-kinetics between O and OT groups remained, even upon adjustment for PCr recovery after exercise (*P* = 0.023), and the differences between O and Y individuals tended to remain significant (*P* = 0.08).

### Skeletal muscle mitochondrial inertia at the onset of exercise is associated with functional outcomes and exercise efficiency.

During the 6-minute walk test (6MWT), both the distance covered and walking speed were not significantly different across the groups (Y, 630 ± 15 m and 1.75 ± 0.104 m/s; OT, 639 ± 17 m and 1.77 ± 0.04 m/s; O, 583 ± 24 m and 1.62 0.06 m/s; *P* = 0.12 for both comparisons; Figure 3D). Furthermore, the chair-stand test did not reveal significant differences between the groups (Y, 9.0 ± 2.5 s; OT, 9.0 ± 1.9 s; O, 10.2 ± 1.6 s; *P* = 0.14; Figure 3E).

Upon performing a submaximal cycling test, gross and net exercise efficiencies were significantly different across the groups (Y, 19.7% ± 0.7% and 23.2% ± 0.90%; OT, 18.3% ± 0.4% and 21.4% ± 0.5 %; O, 15.9% ± 0.4% and 19.0% ± 0.4%; *P* < 0.001 for gross and net efficiency [NE] respectively; Figure 3; F and G). Gross and net exercise efficiencies were significantly lower in O individuals as compared with both Y (*P* < 0.001 for both comparisons) and OT individuals (*P* = 0.003 and *P* = 0.01, respectively). Gross and net exercise efficiencies were not significantly different between Y and OT volunteers (gross efficiency [GE] *P* = 0.23 and NE *P* = 0.11). The Y and OT individuals performed the submaximal cycling test at a similar but significantly higher absolute workload (Y, 109.1 ± 8.4 W; OT, 97. ± 6.8 W; *P* = 0.64) as compared with O (74 ± 6.2 W; *P* < 0.05 for both comparisons). Resting energy expenditure (REE) was not significantly different across the groups (*P* = 0.22; Table 2).

**Figure 4:**
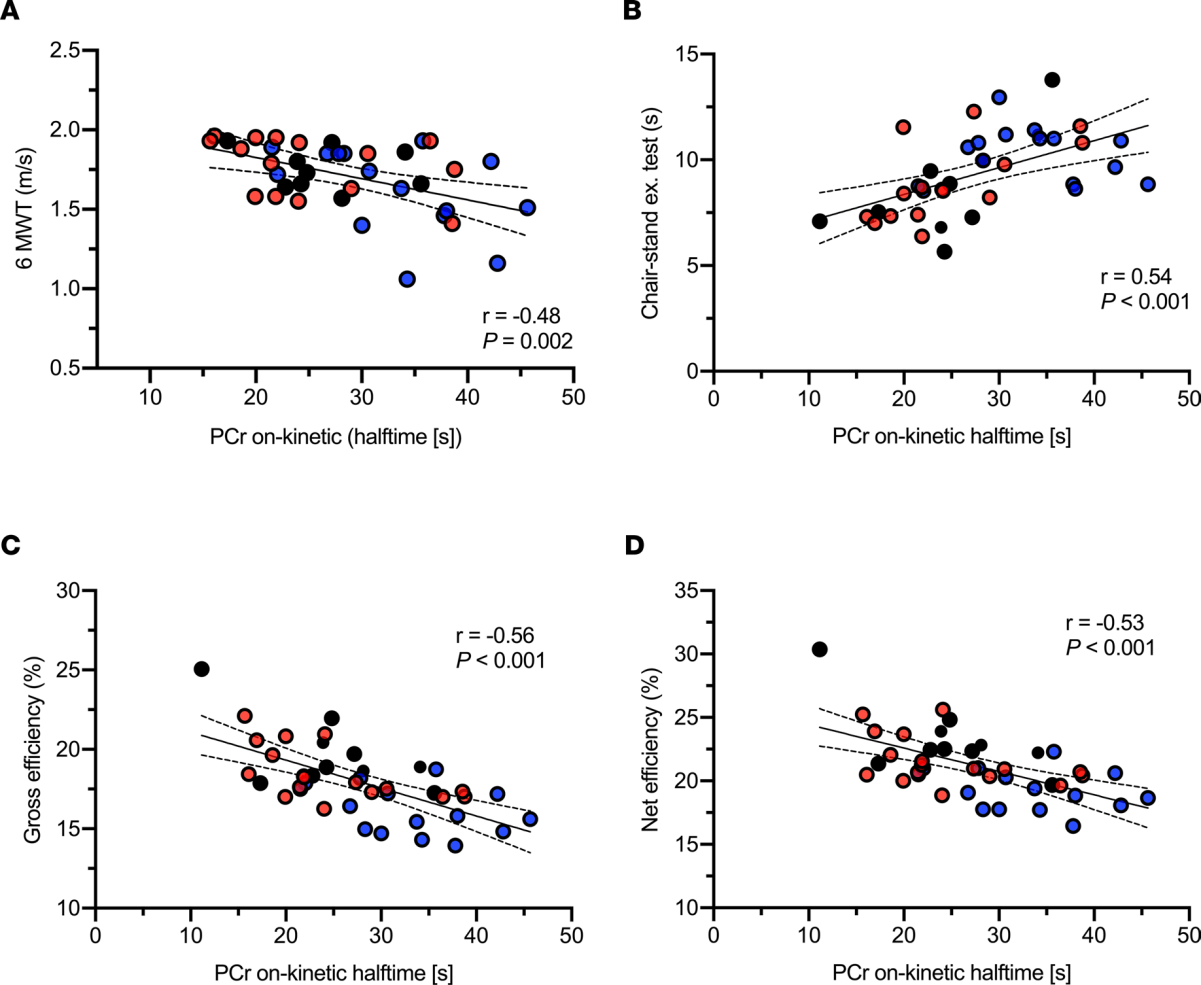
Skeletal muscle mitochondrial inertia is associated with physical function and exercise efficiency in humans. (**A**–**D**) Linear association between PCr on-kinetics and walking speed (**A**), chair seating-standing exercise performance (**B**), gross (**C**), and net exercise efficiency (**D**). [s], seconds; Y, young; OT, older trained; O, older with normal physical activity. Circles in black, Y (*n* = 10); circles in red, OT (*n* = 17); circles in blue, O (*n* = 15). Statistical test was Pearson’s correlation.

Next, we aimed to test if the PCr on-kinetics were related to these physical function parameters. Indeed, we found that slower PCr on-kinetics (mitochondrial inertia) were strongly associated with lower walking speed (*n* = 39, *r* = –0.48, *P* = 0.002; Figure 4A), chair-stand test performance (*n* = 40, *r* = 0.54, *P* < 0.001; Figure 4B), gross exercise efficiency (*n* = 42, *r* = –0.56, *P* < 0.001; Figure 4C), and net exercise efficiency (*n* = 42, *r* = –0.53, *P* < 0.001; Figure 4D). Considering that Y, OT, and O groups differ in terms of age and sex, as well as to investigate whether a slow PCr on-kinetics simply reflects lower mitochondrial function, we recomputed the associations between PCr on-kinetic and the different functional parameters upon adjusting for age, sex, and PCr recovery halftime after exercise (in vivo mitochondrial function). Interestingly, the significant associations of PCr on-kinetic with these functional parameters remained, with the following results: walking speed (*r* = –0.49, *P* = 0.002), chair-stand test performance (*r* = 0.58; *P* < 0.001), gross exercise efficiency (*r* = –0.40; *P* = 0.018), and net exercise efficiency (*r* = –0.34; *P* = 0.046).

## Discussion

Intramyocellular PCr buffers ATP, as the sudden increase in energy demand at the onset of exercise would otherwise lead to ATP depletion. The delay in activating mitochondrial ATP synthesis in response to such sudden increase in ATP demand is known as skeletal muscle mitochondrial inertia. Slower activation of mitochondrial ATP synthesis will result in a more pronounced PCr depletion at the onset of exercise. Assessment of skeletal muscle mitochondrial inertia at the onset of exercise has been hampered by the invasive nature of repeated muscle biopsies. In this cross-sectional study, we applied ^31^P-magnetic resonance spectroscopy (^31^P-MRS) methodology to noninvasively quantify the in vivo PCr kinetics at the onset of exercise (PCr on-kinetics) in 2 different cohorts. It was observed that PCr on-kinetics were significantly slower in older, metabolically compromised volunteers as compared with young, endurance-trained individuals, and they were also significantly slower in older, normally physically active individuals compared with Y individuals and older exercise-trained participants. Moreover, we observed that PCr on-kinetics strongly correlated with multiple markers linked to physical function, such as walking speed, chair sit-to-stand transitions, and mechanical efficiency of exercise. Finally, the observed differences in PCr on-kinetics, and its association with functional outcomes, was strongly associated with CrAT protein activity, acetylcarnitine content, and ADP concentration during exercise in muscle tissue. Collectively, our results support the hypothesis that skeletal muscle mitochondrial inertia is greater in metabolically compromised and older individuals and is closely related to physical function. Furthermore, our findings suggest that a diminished ability of CrAT protein to supply Acetyl-CoA groups for oxidation may underlie skeletal muscle mitochondrial inertia. Alternatively, insensitivity to ADP to stimulate oxidative metabolism may play a role.

Previous investigations from cross-sectional and interventional studies have used similar approaches applying ^31^P-MRS techniques to investigate in vivo PCr on-kinetics in young, healthy volunteers (5, 10, 11) and found that the halftime of PCr on-kinetics is related to the physical fitness of individuals. In fact, the halftime values reported are consistent with our results from T and healthy UT volunteers, as well as our data obtained in young, physically active individuals. However, to the best of our knowledge, no previous studies have specifically examined the PCr kinetics at the onset of exercise in metabolically compromised and older individuals. PCr utilization upon exercise largely depends on the rate of energy supply by mitochondrial OXPHOS (4). Thus, an impaired ability of mitochondria to respond to increased energy demand and to produce ATP might prompt a more prolonged reliance on PCr. This is also referred to as mitochondrial inertia. We show here that PCr on-kinetics are significantly slower in metabolically compromised volunteers and in older individuals with normal physical activity as compared with their young and older trained counterparts. To investigate whether mitochondrial inertia is simply a reflection of mitochondrial capacity, we also adjusted the results for PCr recovery halftime, which is considered a measure of maximal mitochondrial ATP synthetic capacity. Interestingly, the significant differences on PCr on-kinetics remained, even after adjusting by in vivo PCr recovery after exercise. Our results suggest that PCr kinetics at the onset of exercise are a unique characteristic of not yet clearly defined skeletal muscle mitochondrial function, which reflects the readiness of skeletal muscle mitochondria to produce ATP.

To further explore the functional relevance of this yet-unexplored signature of skeletal muscle mitochondrial function, we performed a series of correlative analyses between PCr kinetics at the onset of exercise and various parameters of physical function. PCr kinetics at the onset of exercise proved to be strongly correlated with walking speed and the speed of sit-to-stand transitions, regardless of in vivo skeletal muscle mitochondrial capacity (as determined by PCr recovery after exercise). We investigated the response to a sudden increase on ATP demand, which is typical for the initiation of exercise. A swift response to increased ATP demand can be of particular importance when initiating movements. This supports the contention that skeletal muscle mitochondrial activation is a salient contributor to performance in circumstances that mimic daily-life situations. Hence, PCr kinetics at the onset of exercise might be an important factor of exercise intolerance and may , therefore, be a target of intervention. In this regard, a previous study reported that 5 weeks of regular endurance–type exercise training resulted in an improvement of the PCr kinetics at the onset of exercise, in concert with improving exercise tolerance in young healthy individuals (5).Whether exercise training also improves PCr kinetics at the onset of exercise (mitochondrial activation),and whether it improves muscle functional capacity, in individuals who are prone to premature muscle fatigue warrants future investigation. Furthermore, PCr on-kinetics strongly correlated with exercise efficiency, defined as the ratio between mechanical work and energy expenditure. Interestingly, we observed that PCr on-kinetics correlate with exercise efficiency, even upon correction for skeletal muscle mitochondrial capacity (PCr recovery after exercise). This statistical adjustment suggests a relationship between mitochondrial inertia and functional outcomes, independent of mitochondrial maximal ATP-synthesis rate, and reveals mitochondrial inertia as a signature of mitochondrial function. However, considering the close link between skeletal muscle mitochondrial function and exercise efficiency (12), it is difficult to disentangle the interdependence of PCr on-kinetics and skeletal muscle mitochondrial function. Therefore, the mechanistic link between PCr on-kinetics and exercise efficiency requires further study.

Mechanistically, PCr kinetics at the onset of exercise — hence, skeletal muscle mitochondrial inertia — is governed by the intramyocellular Acetyl-CoA availability (13). Thus, a momentary deficit of Acetyl-CoA groups would restrict the rate of ATP production via oxidative metabolism, thereby causing a prolonged reliance on substrate-level phosphorylation (14). Therefore, we show for the first time to our knowledge in humans that CrAT protein activity in muscle tissue, the enzyme that essentially buffers the intramyocellular Acetyl-CoA content via acetylcarnitine formation and breakdown, is strongly associated with PCr kinetics at the onset of exercise. Furthermore, skeletal muscle acetylcarnitine content was also reduced at rest in metabolically compromised individuals and was significantly associated with slow PCr kinetics at the onset of exercise. In line with the current results, we previously reported, by using a loss-of-function mouse model, that CrAT protein activity prevents the Acetyl-CoA deficit upon exercise via transferring Acetyl-CoA groups from the intracellular acetylcarnitine pool for mitochondrial oxidation (15). We revealed that CrAT-mediated Acetyl-CoA buffering prevents a further reliance on PCr hydrolysis and skeletal muscle glycogen breakdown upon exercise, concluding that CrAT protein function mitigates skeletal muscle mitochondrial inertia and promotes exercise tolerance (15). Other studies reported that elevated Acetyl-CoA/acetylcarnitine content in muscle tissue prior to exercise results in a lower PCr degradation at the onset of exercise and in improved exercise tolerance, independent of increases on muscle blood flow (14, 16). Our findings are consistent with the premise that the stockpiled Acetyl-CoA groups buffered as acetylcarnitine via CrAT protein function are instrumental for skeletal muscle mitochondrial activation, as this might reflect more readily available substrate to fuel the TCA cycle at the onset of exercise. Indeed, we show strong correlations between CrAT protein activity and PCr on-kinetics.

An alternative mechanistic explanation for the observed differences in PCr kinetics across groups at the onset of exercise may be an inherently lower sensitivity of the OXPHOS system to ADP levels to regenerate ATP by active muscles in metabolically challenged groups. Considering the lower intrinsic ex vivo ADP sensitivity previously reported in metabolically compromised (17) and older individuals (18), one might expect that higher ADP levels are needed to stimulate the OXPHOS system in these individuals.

We calculated ADP concentrations, assuming creatine kinase (CK) reaction to be at equilibrium (19) and in line with the notion of ADP sensitivity being of importance; we found higher ADP levels during exercise in patients with T2DM and OB as compared with T individuals. These results suggest that a higher metabolic stress is needed to activate oxidative ATP formation in metabolically compromised individuals, thus relying on substrate-level phosphorylation for a longer duration. The findings regarding the underlying mechanism explaining such differences in sensitivity to metabolic stress remain inconclusive, and next to ADP, other metabolites have been suggested to underlie activation of oxidative metabolism (20). If a stronger reliance on PCr is a contributor to reduced physical function, increasing the PCr pool (e.g., by creatine supplementation) (21, 22) or the acetylcarnitine content (e.g., by carnitine supplementation) (23) in muscle tissue — or even a combined administration — may be beneficial nutritional interventions. Interestingly, a short-term creatine supplementation (5 g/day, for 11 days) increased the intramyocellular PCr pool at rest and during exercise and enhanced ATP resynthesis in young healthy individuals (21). Furthermore, we previously showed that long-term carnitine supplementation (2 g/day, for 36 days) increased the intramyocellular acetylcarnitine levels at rest and the acetylcarnitine formation capacity upon exercise in prediabetic volunteers (23). Nevertheless, it is unknown whether this affects PCr kinetics at the onset of exercise and, eventually, would improve exercise performance.

The use of a noninvasive approach to explore skeletal muscle metabolism during exercise in 2 different cohorts of volunteers is the main strength of the present study. The high time resolution of dynamic ^31^P-MRS allows us to investigate the response of PCr at the onset of exercise in muscle tissue in detail. This allows us to assess the phenomenon of skeletal muscle mitochondrial inertia in metabolically compromised and older individuals. In addition, to explore the potential underlying mechanisms of PCr kinetics at the onset of exercise — hence, skeletal muscle mitochondrial inertia — we also investigated the functional relevance of this phenomenon by measuring PCr kinetics at the onset of exercise along with classical read-outs for functional markers of physical function (6MWT and a chair sit-to-stand test) and exercise efficiency. A limitation of the present study is its cross-sectional nature; therefore, no conclusions can be drawn on whether PCr kinetics at the onset of exercise improves upon exercise training or any other lifestyle intervention. Moreover, the design of the study makes it difficult to disentangle putative age and sex effects: typically, patients with T2DM are of older age and higher BMI than T individuals. Thus, the effects observed in cohort 1 may originate from differences in age, BMI, and metabolic status. The effects observed in cohort 2 suggest that mitochondrial inertia may relate to age and training status without a sec effect: the correlation between PCr on kinetics and markers of physical function remain upon adjustment for sex.

In conclusion, we show that PCr kinetics at the onset of exercise are significantly slower in older, metabolically compromised individuals as compared with young, endurance-trained volunteers, and they are slower in older individuals with normal physical activity as compared with young, healthy and older exercise-trained counterparts, regardless of in vivo skeletal muscle ATP synthetic capacity. Moreover, we report that PCr kinetics at the onset of exercise are a yet-unexplored signature of skeletal muscle mitochondrial metabolism tightly linked to physical function, and they coexist with reduced CrAT activity, low acetylcarnitine levels, and elevated ADP concentration in muscle tissue during exercise. These results indicate that PCr kinetics at the onset of exercise — hence, skeletal muscle mitochondrial inertia — might emerge as a target for intervention to blunt exercise intolerance.

## Methods

### Participants

In this study, we used data from 2 previous studies, which were registered at clinicaltrials.gov with identifiers NCT01298375 (study cohort 1) and NCT03666013 (study cohort 2).

#### Study cohort 1.

As reported previously (9), 38 male volunteers, including 9 older patients with T2DM; 8 older individuals with OB; 9 young, lean, UT volunteers (UT, VO_2_max < 45 mL/kg/min); and 12 young, lean, endurance-trained individuals (T, VO_2_max > 55 mL/kg/min) participated as cohort 1 in the present study. Patients with T2DM and individuals with OB as well as T and UT volunteers were matched for age and BMI.

#### Study cohort 2.

In the current study, we used data from a previous study from individuals in which PCr on-kinetics could be reliably determined. In total, 42 participants including 10 Y, with normal physical activity (20–30 years); 15 O, with normal physical activity (65–80 years); and 17 OT (65–80 years) participants were included in the present study cohort 2.

Individuals were considered normally physically active if they completed no more than 1 structured exercise session per week, while individuals were considered as trained if they engaged in at least 3 structured exercise sessions of at least 1 hour per week for an uninterrupted period of at least the past year.

Participants were excluded from the study if they reported MRI contraindications and if they had a medical history of cardiovascular disease.

### Body composition and maximal aerobic capacity

Body composition in cohort 1 was assessed by dual x-ray absorptiometry (DEXA scan, Hologic Discovery). For study cohort 2, body composition was assessed by air displacement plethysmography (BodPod, COSMED, Inc.) (24). Maximal aerobic capacity (VO_2_max) was determined in both study cohorts by a graded maximal cycling test until exhaustion via indirect calorimetry (Omnical), as described previously (25).

### In vivo skeletal muscle PCr on-kinetics and PCr recovery

All MR measurements were performed on a 3T clinical MRI scanner (Achieva 3T-X Phillips Healthcare) with a 6 cm surface coil. ^31^P-MRS was employed to examine in vivo skeletal muscle metabolism in the m. vastus lateralis during and after exercise, as reported earlier, with a time resolution of 4 seconds (9). To circumvent the influence of exercise intensity on PCr utilization and kinetics, we standardized an exercise protocol to individuals’ maximal capacities, aiming to reach a similar PCr depletion rate in all participants. A knee-extension protocol was performed on a custom-built MRI-compatible ergometer with a pulley system for 5 minutes at 50%–60% of the individuals’ predetermined maximal knee-extension capacity, aiming at PCr depletion of 30%–50% in all subjects. The ^31^P resonances were quantified in MATLAB by peak fitting. The decrease of PCr over time from the onset of exercise to a lower steady state was fitted with a monoexponential function using a custom-written MATLAB script. The halftimes of the fit were used as a parameter of PCr on-kinetics assumed to be a marker of skeletal muscle mitochondrial inertia. Here, a longer halftime indicates a longer reliance on PCr as a source of ATP and, therefore, a more pronounced skeletal mitochondrial inertia (Supplemental Figure 1; supplemental material available online with this article; https://doi.org/10.1172/jci.insight.163855DS1). The time course of the PCr recovery rate post exercise (off-kinetics) was also fitted with a monoexponential function, and the halftime was used as a marker of oxidative capacity, as reported earlier (9). In vivo mitochondrial function is, therefore, expressed as the PCr recovery halftime after exercise, with a shorter halftime indicating a faster recovery and, thus, better mitochondrial function.

### In vivo acetylcarnitine content in skeletal muscle

Skeletal muscle acetylcarnitine content was quantified in volunteers from study cohort 1 by ^1^H-MRS at rest before the ^31^P-MRS protocol, as described earlier (9).

### In vivo ADP levels in skeletal muscle during exercise

The spectra from volunteers of study cohort 1 were additionally fitted in jMRUI by a time domain–fitting routine using the AMARES algorithm (26) in order to calculate ADP levels, assuming CK to be at equilibrium with constant (*K*_CK_ = 1.66 × 10^9^ M^–1^) and upon the assumptions that PCr represents 85% of the total creatine concentration at rest and [ATP] equal to 8.2 mM (27). Resting ADP levels were calculated from the average peak areas (amplitude) of the first 5 dynamic scans (repetition time [TR] = 4 seconds) recorded at rest. In addition, ADP levels at the onset of exercise were computed from the average peak areas (amplitude) of the last 3 dynamic spectra (TR = 4 seconds) before the PCr levels reached a new and lower steady state (Supplemental Figure 1).

### Physical function and exercise efficiency parameters

Functional outcomes were determined in volunteers from cohort 2, who performed the chair-stand exercise test, as previously reported (28), and the 6MWT using the treadmill of the CAREN-system (Computer Assisted Rehabilitation Environment Extended, CAREN; Motekforce Link).

In addition, volunteers from cohort 2 performed a single, submaximal cycling bout of 60 minutes on a cycle ergometer at 50% of their individual maximal power output with oxygen consumption and carbon dioxide production being measured for 15 minutes at minute 15 and minute 45. REE and energy expenditure during steady-state exercise (exercise energy expenditure [EEE]) was calculated using the Weir equation (29). GE was computed as the ratio of power output (watts converted in kJ/min) to EEE during the submaximal cycling test and expressed as percentage as follows:

GE (%) = (Work _(kJ/min)_/EEE _(kJ/min)_ × 100

NE was computed as the ratio of power output (watts converted to kJ/min) to EEE minus REE and expressed as percentage as previously described (30):

NE (%) = (Work _(kJ/min)_/(EEE _(kJ/min)_ – REE _(kJ/min)_) × 100

### Whole-body insulin sensitivity and muscle biopsy

For the characterization of the metabolically compromised individuals included in the present study, we here report the outcomes of whole-body insulin sensitivity from individuals of study cohort 1 who underwent a 2-step, hyperinsulinemic-euglycemic clamp (10 and 40 mU/kg/min), as originally described (31). Before starting the glucose and insulin infusion, a muscle biopsy was taken from the m. vastus lateralis under local anesthesia (2% lidocaine), directly frozen in melting isopentane, and stored at –80°C until further analysis. CrAT protein activity was assessed using 0.01 mg of soluble protein lysate in 50 mM Tris (pH 7.4), 1 mM EDTA, 0.1M DTNB, 1.0 mM Acetyl-CoA, and 5 mM L-carnitine at 25°C (9).

### Statistics

Participant characteristics are expressed as mean ± SD, while all other results are expressed as mean ± SEM. Statistical analysis was performed using SPSS, version 21.0 (IBM Corp.). Shapiro-Wilk normality test was carried out to evaluate normal distribution. A 1-way ANOVA with Bonferroni post hoc correction was used to test for statistical differences across groups for participant characteristics and outcome parameters in both study cohorts. Further comparisons of the differences in our primary outcome (PCr on-kinetic) across groups in both study cohorts were conducted using 1-way ANCOVA, implementing the PCr recovery rate after exercise as a covariate. Sex distribution across the groups of study cohort 2 was determined by χ^2^ test. To test for significant linear association between variables by using individual data, we conducted bivariate Pearson’s correlation and partial correlation analyses corrected for PCr recovery rate after exercise, age, and sex. In all tests, *P* < 0.05 was set to be statistically significant.

### Study approval

This study was approved by the medical Ethical Committee of the Maastricht University Medical Center. All individuals gave written informed consent before enrollment, and the study was conducted in accordance with the Declaration of Helsinki.

## Author contributions

RFM, JH, PS, VS, and MKCH, conceived and designed the study. RFM, LL, LG, TRK, DMM, JH, PS, VSH, and MKCH analyzed and interpreted the data. RM, VS, and MH wrote the manuscript. RFM and MKCH revised and approved the final version of the manuscript. All authors reviewed and approved the final version of the manuscript. MKCH is the guarantor of this work and, as such, had full access to all the data in the study and takes responsibility for the integrity of the data and the accuracy of the data analysis.

## Supplementary Material

ICMJE disclosure forms

Supplemental figure 1

## Figures and Tables

**Table 1:**
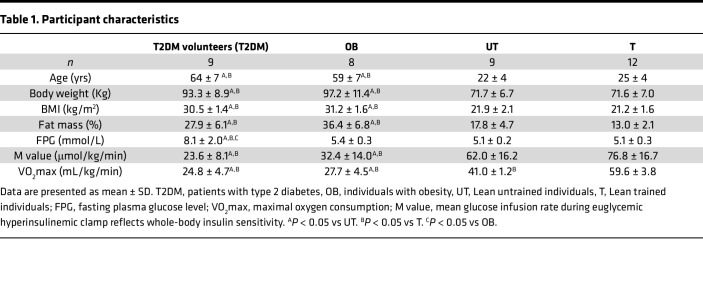
Participant characteristics

**Table 2:**
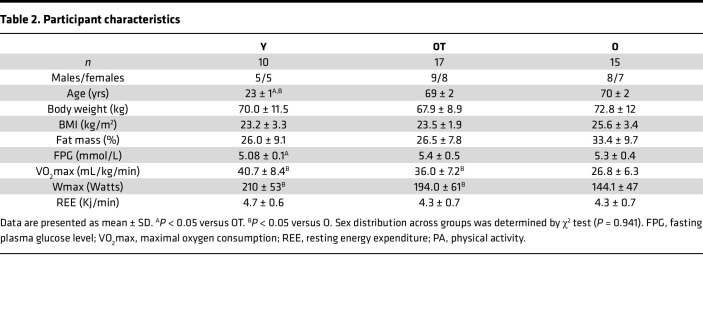
Participant characteristics

## Version 1 11/22/2022

In-Press Preview

## Version 2 01/10/2023

Electronic publication
