# Supplementary material for: Skeletal muscle mitochondrial inertia is associated with carnitine acetyltransferase activity and physical function in humans
Source: JCI Insight. 2023 Jan 10;8(1):e163855. doi: 10.1172/jci.insight.163855 (PMC9870054; doi:10.1172/jci.insight.163855)

Supplemental Figure 1. Time course of PCr in muscle during and after the knee-extension exercise assessed by ^{31}P-MRS. Red line indicates PCr on-kinetics towards a lower steady state during exercise, which was fitted with a mono-exponential function (green line). The half-time [s] of the fit was used as a parameter of PCr on-kinetics assumed to be a marker of skeletal muscle mitochondrial inertia.

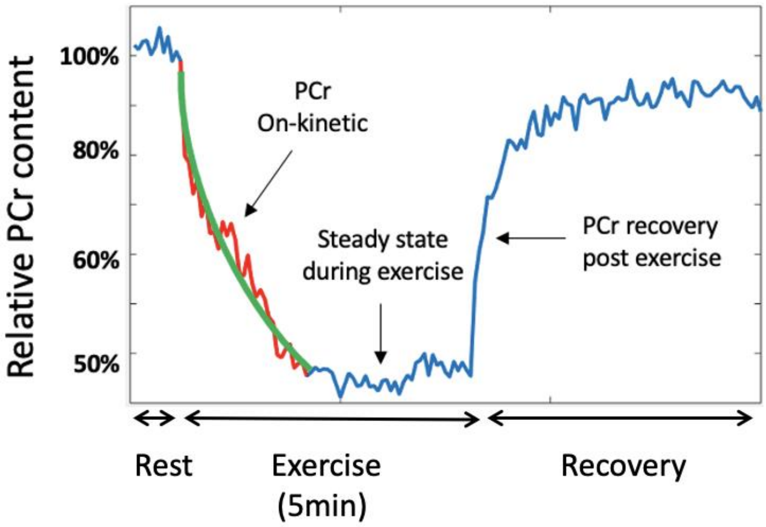

Supplement: Supplemental figure 1 [file jciinsight-8-163855-s222.pdf]
